# CRISPR-Cas9 Library Screening Identifies Novel Molecular Vulnerabilities in *KMT2A*-Rearranged Acute Lymphoblastic Leukemia

**DOI:** 10.3390/ijms241713207

**Published:** 2023-08-25

**Authors:** Pauline Schneider, Priscilla Wander, Susan T. C. J. M. Arentsen-Peters, Kirsten S. Vrenken, Dedeke Rockx-Brouwer, Fabienne R. S. Adriaanse, Veerle Hoeve, Irene Paassen, Jarno Drost, Rob Pieters, Ronald W. Stam

**Affiliations:** 1 Princess Máxima Center for Pediatric Oncology, 3584 CS Utrecht, The Netherlands; 2 Oncode Institute, 3521 AL Utrecht, The Netherlands

**Keywords:** *KMT2A*-rearranged, infant, leukemia, CRISPR-Cas9, epigenome, kinome, ARID4B, MBD3, BMPR2

## Abstract

In acute lymphoblastic leukemia (ALL), chromosomal translocations involving the *KMT2A* gene represent highly unfavorable prognostic factors and most commonly occur in patients less than 1 year of age. Rearrangements of the *KMT2A* gene drive epigenetic changes that lead to aberrant gene expression profiles that strongly favor leukemia development. Apart from this genetic lesion, the mutational landscape of *KMT2A*-rearranged ALL is remarkably silent, providing limited insights for the development of targeted therapy. Consequently, identifying potential therapeutic targets often relies on differential gene expression, yet the inhibition of these genes has rarely translated into successful therapeutic strategies. Therefore, we performed CRISPR-Cas9 knock-out screens to search for genetic dependencies in *KMT2A*-rearranged ALL. We utilized small-guide RNA libraries directed against the entire human epigenome and kinome in various *KMT2A*-rearranged ALL, as well as wild-type *KMT2A* ALL cell line models. This screening approach led to the discovery of the epigenetic regulators *ARID4B* and *MBD3*, as well as the receptor kinase *BMPR2* as novel molecular vulnerabilities and attractive therapeutic targets in *KMT2A*-rearranged ALL.

## 1. Introduction

Acute lymphoblastic leukemia (ALL) represents the most common type of cancer diagnosed in children and is currently curable in ~90% of patients [1]. Unfortunately, the survival chances for infants with ALL, patients <1 year of age, are significantly worse. Overall, the event-free survival (EFS) chances for infants diagnosed with ALL are ~50% [2,3]. Approximately 80% of the cases of infant ALL are characterized by chromosomal translocations involving the Lysine Methyltransferase 2A (*KMT2A*) gene in chromosome 11q23, in which the N-terminus of *KMT2A* fuses with the C-terminus of one of its translocation partner genes, such as *AFF1* (*AF4*; in ~49% of cases), *MLLT1* (*ENL*; ~22%) or *MLLT3* (*AF9*; ~16%) [4]. Strikingly, the 6-year EFS chances for *KMT2A*-rearranged infant ALL patients are at best 40% [2,3]. Hence, currently available treatment regimens clearly do not suffice and finding more effective therapeutic strategies still represents an unmet but urgent clinical need.

Functionally, wild-type *KMT2A* plays an essential role in regulating gene expression during early development and hematopoiesis [5] regulating gene transcription through histone 3 lysine 4 (H3K4) methyltransferase activity mediated by its C-terminal Su(Var)3–9, Enhancer-of-zeste, and Trithorax (SET) domain [6,7,8]. In contrast, oncogenic KMT2A fusion proteins lose the SET domain, but instead recruit the histone methyltransferase DOT1L, which catalyzes the dimethylation of histone H3 on lysine 79 (H3K79me2) [6,9,10], leading to aberrant gene expression profiles that strongly favor leukemogenesis [11,12].

In addition to the use of immunotherapeutic approaches such as blinatumomab [13], treatment of *KMT2A*-rearranged ALL (KMT2A-r ALL) may be improved by using epigenetic-based drugs targeting epigenetic vulnerabilities that are specifically essential to this type of leukemia. For instance, we recently showed that *KMT2A*-r ALL responds remarkably well to histone deacetylase (HDAC) inhibition [14,15]. Interestingly, small-molecule kinase inhibitors often exhibit synergistic anticancer effects in combination with HDAC inhibition, which led to the development of a rapidly expanding repertoire of chimeric HDAC/kinase dual inhibitors [16,17]. Moreover, *FLT3*, a receptor tyrosine kinase, has been previously identified as a vulnerability in *KMT2A*-r ALL. A recent clinical trial with the FLT3 inhibitor lestaurtinib revealed that patients whose leukemia blasts exhibited sensitivity to FLT3 inhibition ex vivo experienced benefits from the addition of the FLT3 inhibitor to chemotherapy [18]. Given that kinases represent the largest group of druggable targets in the human genome [19], and that *KMT2A*-r ALL is an epigenetically driven malignancy, combinations of epigenetic-based drugs and kinase inhibitors may well represent effective treatments for this elusive type of leukemia.

Therefore, we set out to identify novel epigenetic regulators and kinases specifically essential to *KMT2A*-r ALL cells by applying in vitro clustered regularly interspaced short palindromic repeats (CRISPR)-associated protein 9 (Cas9) knockout screens using synthetically designed single guide RNA (sgRNA) libraries [20,21] directed against the entire human epigenome and kinome [22,23]. CRISPR-Cas9 technology has had a major impact on drug (target) discovery and development due to its ability to efficiently altering genomic information in mammalian cells [24]. In the present study this approach led to the identification of known as well as novel molecular vulnerabilities and potential therapeutic targets in *KMT2A*-r ALL.

## 2. Results

### 2.1. CRISPR-Cas9 Knockout Screens in KMT2A-Rearranged and Wild-Type KMT2A ALL Cells

**Figure 1:**
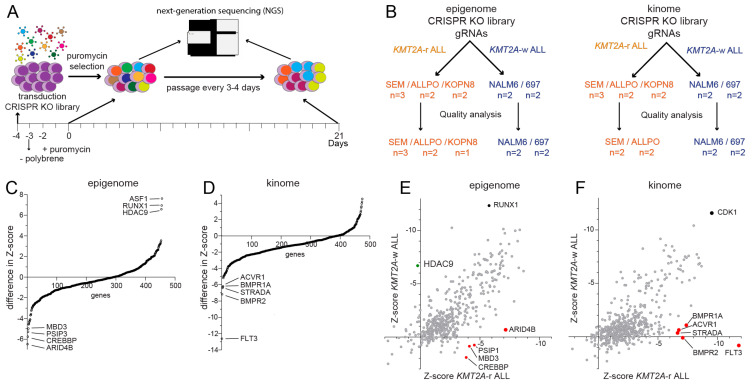
**Epigenome and kinome CRISPR knockout screen identifies epigenetic genes and druggable targets essential for *KMT2A*-rearranged ALL cells.** (**A**) Graphic overview of the CRISPR-Cas9 knockout (KO) screen. (**B**) Schematic overview of the CRISPR KO screen samples. The KMT2A-r ALL cell lines are marked in orange and the KMT2A-w ALL cell lines in blue. (**C**,**D**) Graph showing the difference in Z-scores, calculated by the MLE module of MAGeCK, for genes targeted in the epigenome CRISPR KO screen (**C**) and the kinome CRISPR KO screen (**D**) between *KMT2A*-r ALL cell lines and *KMT2A*-w ALL cell lines. (**E**,**F**) Graphs showing the average Z scores of genes targeted in the epigenome (**E**) and the kinome CRISPR KO screen (**F**) for the *KMT2A*-r ALL cell lines and *KMT2A*-w ALL cell lines plotted against each other. The red dots represent specific vulnerabilities in KMT2A-r ALL, the green dot represents a specific vulnerability in KMT2A-w ALL.

To identify epigenetic regulators and kinases essential for leukemia proliferation, maintenance, and survival, we conducted CRISPR-Cas9 knockout screens using sgRNA libraries targeting the human epigenome (446 genes) and kinome (504 genes). To distinguish between genes that are specifically essential to *KMT2A*-r ALL and those essential for ALL in general, we performed screens in *KMT2A*-r ALL cell lines (i.e., SEM, ALL-PO, and KOPN-8) as well as wild-type *KMT2A* (*KMT2A*-w) B-cell precursor (BCP) ALL cell lines (i.e., NALM-6 and 697). The experimental outline (Figure 1A) is based on a study by Shalem et al., in which sgRNAs were delivered into leukemia cells through lentiviral transduction, and non-transduced cells were eliminated through puromycin selection [21]. Samples drawn on day 0 provided the baseline representation of the sgRNA libraries, and samples drawn on day 21 were used to determine which of the sgRNAs were lost from the leukemic cell populations as a result of targeting genes essential to the proliferation and/or viability of the cells. For all cell line models, we obtained high-quality data for at least two independent replicates, except for KOPN-8, for which only a single sample provided reliable data in the epigenome screen (Figure 1B).

Approximately 67% or 76% of the sequenced reads at baseline (day 0) could be mapped to the epigenome or kinome sgRNA library, respectively. Read counts for non-targeting control sgRNAs remained stable between day 0 and day 21, indicating that the transduction of non-targeting sgRNAs did not affect cell viability or proliferation (Figure S1). Read counts for sgRNAs directed against genes known to be essential to human cells in general (i.e., positive control sgRNAs) markedly decreased over time (Figure S1) indicating that sgRNAs directed against essential genes indeed disappear from the leukemic cell populations.

### 2.2. Identification of Novel Epigenetic Regulators and Kinases Specifically Essential to KMT2A-r ALL

**Figure 2:**
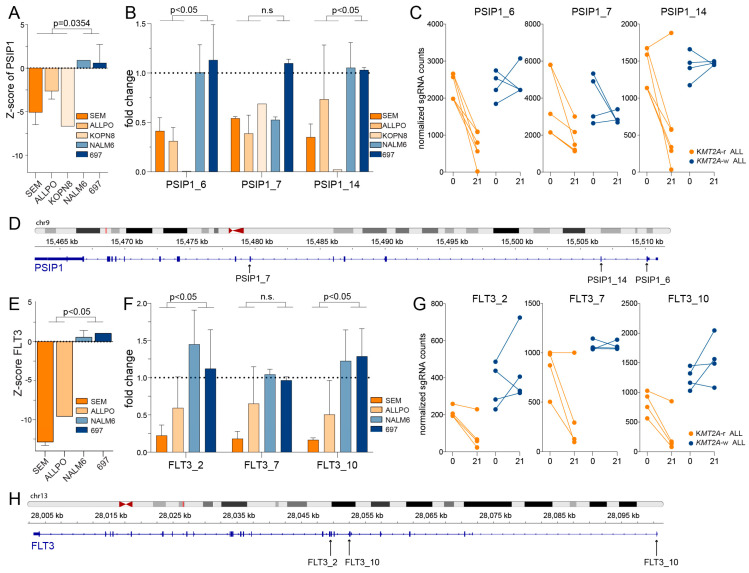
**Evaluation of the known *KMT2A*-rearranged ALL vulnerability genes PSIP1 and FLT3.** (**A**) The z-scores of individual cell lines for *PSIP1* knockout. (**B**) The fold change of normalized read counts on day 21 compared to baseline (day 0) for sgRNAs PSIP1_6, PSIP1_7, and PSIP1_14 derived from our original screening libraries. The mean value with the standard error of the mean (SEM) is depicted from two independent experiments. (**C**) The normalized read counts on day 0 and day 21 of these sgRNAs. (**D**) Overview of the target locations on the *PSIP1* gene for the sgRNAs presented in (**A**,**B**). (**E**) The z-scores of individual cell lines for *FLT3* knockout. (**F**) The fold change of normalized read counts on day 21 compared to baseline (day 0) for sgRNAs FLT3_2, FLT3_7 and FLT3_10 derived from our original screening libraries depicted for two independent experiments +/− SEM. (**G**) The normalized read counts on day 0 and day 21 of these sgRNAs are presented by the mean +/− SEM of two independent experiments. (**H**) Overview of the target locations of the sgRNAs presented in (**F**,**G**) on the *FLT3* gene. All differences in the figure were statistically evaluated using unpaired *t*-tests.

First, we determined the difference in z-scores between *KMT2A*-r ALL cell lines against those of *KMT2A*-w ALL cell lines (Figure 1C,D). We identified *ARID4B*, *CREBBP*, *PSIP1* and *MBD3* as epigenetic regulator genes more essential to *KMT2A*-r ALL, and *ASF1*, *RUNX1* and *HDAC9* as more essential to *KMT2A*-w ALL (Figure 1C). In addition, we found *FLT3*, *BMPR2*, *STRADA*, *BMPR1A*, and *ACVR1* to represent kinases most essential to *KMT2A*-r ALL cells (Figure 1D). Next, we plotted the average z-scores of the *KMT2A*-r ALL cell line models against those of the *KMT2A*-w ALL cell lines (Figure 1E,F). This revealed that *RUNX1* is an essential epigenetic regulator in both *KMT2A*-r and *KMT2A*-w BCP-ALL, which is consistent with previously published data [25,26,27,28,29,30,31,32]. Among the genes specifically essential for the survival and proliferation of *KMT2A*-r ALL cells we found *PSIP1* and *CREBBP* (Figure 1E), as well as *FLT3* (Figure 1F), representing known vulnerabilities in *KMT2A*-r ALL [33,34,35,36,37,38,39,40,41,42]. *PSIP1*, also known as LEDGF/p75, plays a vital role in chromatin organization and transcriptional regulation [33,34]. *CREBBP*, a transcriptional coactivator, is involved in acetylating histones and regulating gene expression [35]. *FLT3*, a receptor tyrosine kinase, has previously been identified as a vulnerability in *KMT2A*-r ALL [38,39]. For all three genes, the z-scores were significantly lower in *KMT2A*-r ALL cell lines as compared with *KMT2A*-w BCP-ALL cell lines (Figure 2A,E and Figure S2A) as were the read counts for individual sgRNA sequences on day 21 (Figure 2B–D,F–H and Figure S2B–D). These results underscored the known importance of *PSIP1*, *CREBBP*, and *FLT3*, as the knockout of these genes resulted in impaired cell growth and survival specifically in *KMT2A*-r ALL cells, indicating their crucial roles. These observations clearly emphasize the validity of our screens and the newly identified genetic dependencies in *KMT2A*-r ALL, including the epigenetic regulators *MBD3* and *ARID4B* (Figure 1C,E), and the kinases *BMPR2, ACVR1, BMPR1A*, and *STRADA* (Figure 1D,F). Considering that *BMPR2*, *BMPR1A*, and *ACVR1* are all receptors for bone morphogenetic proteins (BMPs), we have selected the receptor that exhibits the most differential response between *KMT2A*-r and *KMT2A*-w ALL cells for further validation, i.e., *BMPR2*.

### 2.3. Validation of ARID4B and MBD3 as Epigenetic Dependencies in KMT2A-r ALL Cells

**Figure 3:**
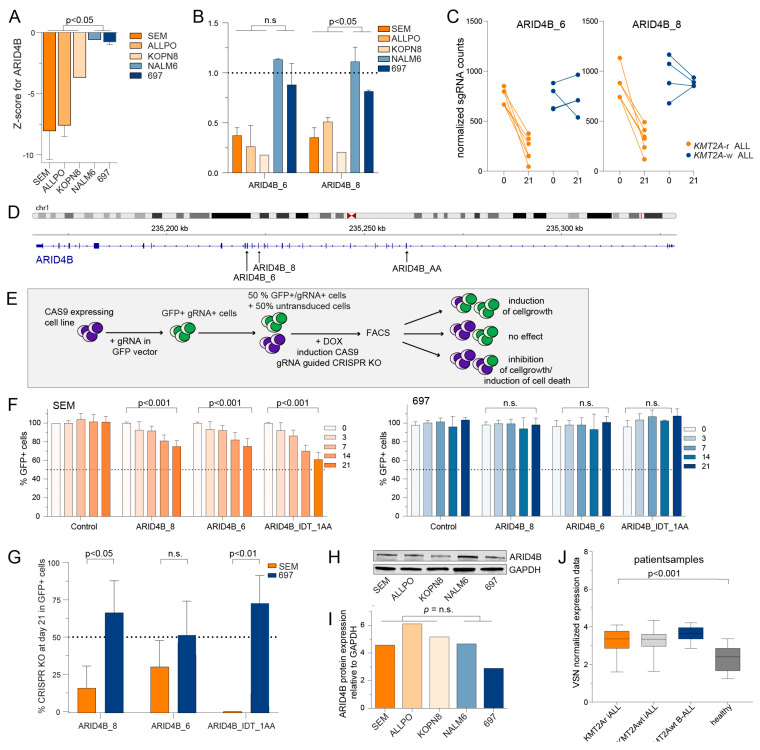
**Validation of novel identified epigenetic regulator *ARID4B* implicated in *KMT2A*-r ALL**. (**A**) The z-scores of individual cell lines for *ARID4B* knockout. Differences were statistically evaluated using unpaired *t*-tests. (**B**) The fold change of normalized read counts on day 21 compared to baseline (day 0) for sgRNAs ARID4B_6 and ARID4B_8 derived from our original screening libraries. The mean value +/− the SEM is depicted from two independent experiments from the CRISPR KO screen. Differences were statistically evaluated using unpaired *t*-tests. (**C**) The normalized read counts on day 0 and day 21 of these sgRNAs. (**D**) Overview of the target locations on the *ARID4B* gene of the sgRNAs used for validation experiments. (**E**) graphic overview of the green fluorescent protein (GFP) competition assay. (**F**) The percentages of GFP positive cells in the GFP competition assay for sgRNAs targeting *ARID4B* in *KMT2A*-r ALL cell line SEM and *KMT2A*-w ALL cell line 697 were measured by flow cytometry. The proportion of GFP positive cells was normalized to the GFP positive cells at baseline (day 0). The data represent the mean +/− SEM of two independent experiments and the differences were statistically evaluated using multiple *t*-test. (**G**) Percentages of CRISPR KO score as determined by sequencing data analysis using the Synthego ICE Analysis tool for sgRNAs targeting *ARID4B* in *KMT2A*-r ALL cell line SEM and *KMT2A*-w ALL cell line 697. The data represent the mean of two or three sequencing analysis +/− the SEM. (**H**) Immunoblot images of ARID4B and GAPDH protein levels in *KMT2A*-r ALL cell lines SEM, ALL-PO, KOPN8, and *KMT2A*-w ALL cell lines NALM6 and 697. (**I**) Protein expression quantification of immunoblot (**H**) by densitometry analysis of ARID4B compared to GAPDH (**J**) VSN normalised microarray data (Affymetrix HU133plus2.0 GeneChips) showing the expression of *ARID4B* (probeset 224322_at), in infant ALL patients carrying *KMT2A* translocations (i.e., *KMT2A*r iALL, *n* = 59), infant ALL patients without *KMT2A* translocations (i.e., *KMT2A*wt iALL, *n* = 14) and childhood ALL patients older than 1 year of age without *KMT2A* translocations (i.e., *KMT2A*wt BALL, *n* = 16) and healthy bone marrow samples (i.e., healthy, *n* = 13). Data depicted in box and whiskers plots with minimum and maximum values.

**Figure 4:**
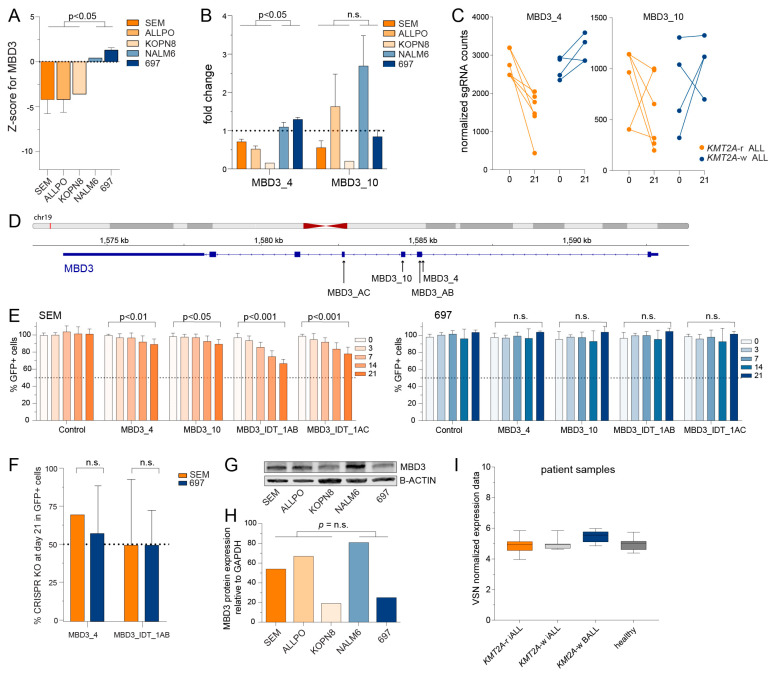
**Validation of novel identified epigenetic regulator *MBD3* implicated in *KMT2A*-r ALL**. (**A**) The z-scores of the individual cell lines for *MBD3* knockout. Differences were statistically evaluated using unpaired *t*-tests. (**B**) The fold change of normalized read counts on day 21 compared to baseline (day 0) for sgRNAs MBD3_4 and MBD3_10 derived from our original screening libraries. The mean value +/- the SEM is depicted from two independent experiments from the CRISPR KO screen. Differences were statistically evaluated using unpaired *t*-tests. (**C**) The normalized read counts on day 0 and day 21 of these sgRNAs. (**D**) Overview of the target locations on the *MBD3* gene of the sgRNAs used for validation experiments. (**E**) The percentages of GFP positive cells in the GFP competition assay for sgRNAs targeting *MBD3* in *KMT2A*-r ALL cell line SEM and *KMT2A*-w ALL cell line 697 were measured by flow cytometry. The proportion of GFP positive cells was normalized to the GFP positive cells at baseline (day 0). The data represent the mean +/− SEM of two independent experiments and the differences were statistically evaluated using multiple *t*-test. (**F**) Percentages of CRISPR KO score as determined by sequencing data analysis using the Synthego ICE Analysis tool for sgRNAs targeting *MBD3* in *KMT2A*-r ALL cell line SEM and *KMT2A*-w ALL cell line 697. The data represent the mean of two or three sequencing analysis +/− the SEM. (**G**). Immunoblot images of MBD3 and B-ACTIN protein levels in *KMT2A*-r ALL cell line SEM and *KMT2A*-w ALL cell line 697. The data represent the mean of two or three sequencing analysis +/− the SEM. (**H**) Protein expression quantification of immunoblot (**H**) by densitometry analysis of MBD3 compared to B-ACTIN. (**I**) VSN normalised microarray data (Affymetrix HU133plus2.0 GeneChips) showing the expression of MBD3 (probeset 41160_at), in infant ALL patients carrying *KMT2A* translocations (i.e., *KMT2A*r iALL, *n* = 59), infant ALL patients without *KMT2A* translocations (i.e., *KMT2A*wt iALL, *n* = 14) and childhood ALL patients older than 1 year of age without *KMT2A* translocations (i.e., *KMT2A*wt BALL, *n* = 16), and healthy bone marrow samples (i.e., healthy, *n* = 13). Data depicted in box and whiskers plots with minimum and maximum values.

For both *ARID4B* and *MBD3*, the z-scores were consistently and significantly lower in all *KMT2A*-r ALL cell line models (Figure 3A and Figure 4A), as were the read counts of individual sgRNA sequences (Figure 3B–D and Figure 4B–D). To validate whether *ARID4B* and *MBD3* truly represent novel molecular vulnerabilities in *KMT2A*-r ALL, we used a GFP-based competition assay, recently described as a powerful screening methodology to identify novel therapeutic targets [43,44]. This assay involves the mixing of cells transduced with doxycycline (Dox)-inducible sgRNA/GFP expression vectors with non-transduced cells in equal proportions and monitoring the levels of GFP + cells over time using flow cytometry (Figure 3E). These competition assays were performed in the *KMT2A*-r ALL cell lines SEM and in the *KMT2A*-w BCP-ALL cell lines 697, using both sgRNAs derived from our original screening libraries as well as with commercially available sgRNAs with high efficiency and low off-target effects (i.e., ARID4B_IDT_1AA, MBD3_AB, and MBD3_AC). The location of these sgRNA sequences in *ARID4B* and *MBD3*, respectively, are indicated in Figure 3D and Figure 4D. For all tested sgRNAs directed against either ARID4B or MBD3, the number of GFP^+^ leukemic cells was progressively and significantly reduced over time in *KMT2A*-r ALL cells line SEM, but not in *KMT2A*-w ALL cell line 697 (Figure 3F and Figure 4E).

To determine whether the observed decreases in the GFP^+^ leukemic cell population was due to an effect on cell viability or cell proliferation, we evaluated the efficiency of CRISPR editing by measuring the percentage of cells within the GFP^+^ cells that exhibited insertions or deletions on day 21 as determined by sequence analysis. This measurement allowed us to determine a CRISPR Knockout Score (KO Score) using the Synthego ICE Analysis tool. When genes that are essential for cell viability are targeted, knockout leads to cell death accompanied by the loss of the sgRNA sequence from the cell pool. In the case of *ARID4B*, all tested sgRNAs showed low KO scores ranging from 1% to 35% in the *KMT2A*-r ALL cell line SEM, with the commercially available sgRNA (i.e., ARID4B_IDT_1AA) producing the lowest score (Figure 3G). In contrast, the scores in the *KMT2A*-w BCP-ALL cell line 697 remained consistently high for all sgRNAs. Taken together, this suggests that *ARID4B* is essential for leukemic cell survival in *KMT2A*-r ALL but not in *KMT2A*-w BCP-ALL.

Interestingly, the scores for *MBD3* remained high in both SEM and 697 cells, indicating that the sgRNA sequences directed against *MBD3* were still present in both cell line models (Figure 4F). These findings suggest that the significant decrease in the percentage of GFP^+^ SEM cells could not have been the result of leukemic cell death, but instead is more likely due to inhibition of cell proliferation.

Since *ARID4B* overexpression was found to be of prognostic value in other types of cancer [45,46,47], we explored the protein expression of ARID4B in all cell lines used in our CRISPR-Cas9 knockout screens, and found no significant differences (Figure 3H,I). Comparison of transcriptional levels of patient samples diagnosed with either *KMT2A*-r ALL or *KMT2A*-w ALL, as well as healthy bone marrow samples, retrieved from previously performed expression arrays [12], also revealed no differences in expression between leukemia types, although the overall expression of *ARID4B* in pediatric ALL was significantly higher than in healthy bone marrow cells (Figure 3J). Likewise, there were no notable differences in *MBD3* expression between *KMT2A*-r ALL and *KMT2A*-w BCP-ALL (Figure 4G–I). Hence the specific dependency of *KMT2A*-r ALL cells on *ARID4B* and *MBD3* is not caused by increased levels of expression.

### 2.4. Validation of Receptor Kinase BMPR2 as a Molecular Vulnerability in KMT2A-r ALL Cells

**Figure 5:**
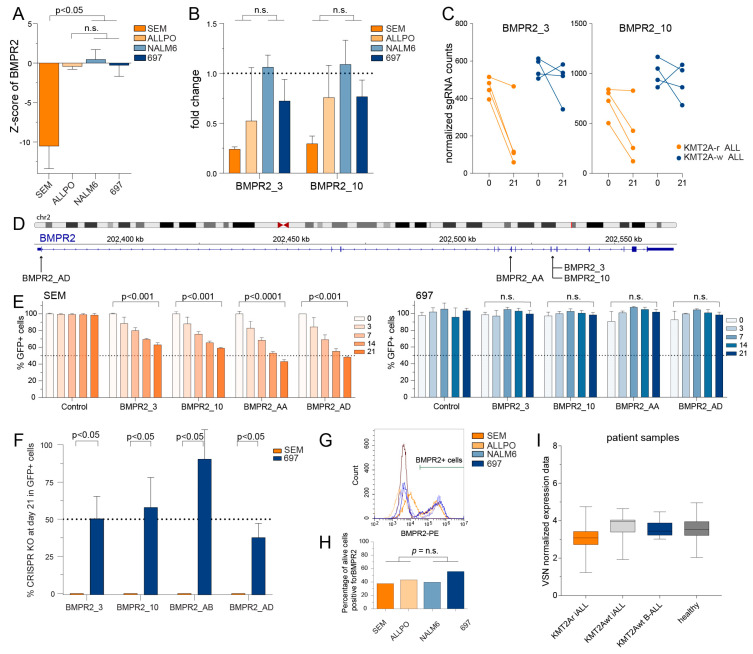
**Validation of novel identified kinase *BMPR2* essential for *KMT2A*-rearranged ALL cells**. (**A**) The z-scores of the individual cell lines for *BMPR2* knockout. (**B**) The fold change of normalized read counts on day 21 compared to baseline (day 0) for sgRNAs BMPR2_3 and BMPR2_10 derived from our original screening libraries. The mean value with the standard error of the mean (SEM) is depicted from two independent experiments from the CRISPR KO screen. Differences were statistically evaluated using unpaired *t*-tests. (**C**) The normalized read counts on day 0 and day 21 of these sgRNAs. (**D**) Overview of the target locations on the *BMPR2* gene of the sgRNAs used for validation experiments. (**E**) The percentages of GFP positive cells in the GFP competition assay for sgRNAs targeting *BMPR2* in *KMT2A*-r ALL cell line SEM and *KMT2A*-w ALL cell line 697 were measured by flow cytometry. The proportion of GFP positive cells was normalized to the GFP positive cells at baseline (day 0). The data represent the mean with SEM of two independent experiments and the differences were statistically evaluated using multiple *t*-test. (**F**) Percentages of CRISPR KO score as determined by sequencing data analysis using the Synthego ICE Analysis tool for sgRNAs targeting *BMPR2* in *KMT2A*-r ALL cell line SEM and *KMT2A*-w ALL cell line 697. The data represent the mean of two or three sequencing analysis with the SEM. (**G**) Flow cytometry analysis of live cells positive for BMPR2 in *KMT2A*-r ALL cell lines SEM and ALL-PO, and *KMT2A*-w ALL cell line NALM6 and 697. (**H**) Quantification of Flow cytometry analysis of BMPR2 positive cells. The data represent the mean of two Flow cytometry experiments with the SEM. (**I**) VSN normalised microarray data (Affymetrix HU133plus2.0 GeneChips) showing the expression of BMPR2 (probeset 225144_at), in infant ALL patients carrying *KMT2A* translocations (i.e., *KMT2A*r iALL, *n* = 59), infant ALL patients without *KMT2A* translocations (i.e., *KMT2A*wt iALL, *n* = 14) and childhood ALL patients older than 1 year of age without *KMT2A* translocations (i.e., *KMT2A*wt BALL, *n* = 16) and healthy bone marrow samples (i.e., healthy, *n* = 13). Data depicted in box and whiskers plots with minimum and maximum values.

Our CRISPR-Cas9 knockout screens using the sgRNA library directed against the human kinome identified the *BMPR2* as an essential gene to *KMT2A*-r ALL cells. However, our z-score analysis showed that the *KMT2A*-r ALL cell line SEM, but not ALL-PO, had a significantly decreased z-score (Figure 5A). Read count analysis for individual sgRNA sequences directed against *BMPR2* revealed that for the ALL-PO cell line, one of the duplicates clearly showed a decreased expression of the sgRNA sequence, while the expression in the other duplicate remained stable over time (Figure 5B–D).

Validation experiments using the competition assay (see above) revealed a significant loss of GFP signal in the *KMT2A*-r ALL cell line SEM, but not in the *KMT2A*-w BCP-ALL cell line 697 (Figure 5E). Moreover, the CRISPR KO scores clearly demonstrate that all sgRNA tested disappeared from the leukemic cell population in *KMT2A*-r ALL SEM cells, while clearly remaining present in *KMT2A*-w BCP-ALL 697 cells (Figure 5F). Hence, knockout of *BMPR2* appears to be lethal to *KMT2A*-r ALL cells, while *KMT2A*-w BCP-ALL cells remain viable upon losing *BMPR2*. Again, the remarkable dependency of *KMT2A*-r ALL cells on *BMPR2* does not seem to be a consequence of differential *BMPR2* expression as we observed no differences in neither the protein nor the mRNA expression levels between *KMT2A*-r ALL and *KMT2A*-w BCP-ALL (Figure 5G–I).

## 3. Discussion

In this study we used CRISPR-Cas9 drop-out screens using sgRNA libraries against the human epigenome and kinome in various cell line models to identify novel molecular vulnerabilities in *KMT2A*-r ALL. These efforts and additional validation experiments revealed *PSIP1*, *CREBBP,* and kinase *FLT3* representing known vulnerabilities in *KMT2A*-r, as well as *ARID4B*, *MBD3*, and *BMPR2* as potential candidates to be considered as novel therapeutic targets in this aggressive type of leukemia. While the selected cell lines may not be a complete representation of *KMT2A*-r and *KMT2A*-w ALL, it is important to note that this approach successfully pinpointed well-established vulnerabilities specific to *KMT2A*-r ALL, such as *CREBBP*, *PSIP1*, and *FLT3*. These findings align with existing knowledge about the involvement of these genes in *KMT2A-*r ALL, supporting the reliability of our approach in detecting relevant targets.

As shown, loss of *ARID4B*, encoding AT-Rich Interaction Domain 4B (a member of the ARID family of chromatin remodeling proteins), is specifically lethal to *KMT2A*-r ALL cells. *ARID4B* plays a role in various cellular processes including embryonic development, cell proliferation, differentiation, and apoptosis [48,49,50,51]. Apart from this, several studies demonstrated that *ARID4B* plays a significant role in cancer development, metastasis, and cancer-related signaling pathways in different types of human cancers, including breast cancer, ovarian cancer, and hepatocellular carcinoma [45,46,47,52,53]. It functions as a component of the SIN3A transcriptional corepressor complex, which is dependent on histone deacetylase activity and is involved in regulating gene expression [54,55]. With its AT-rich domain, *ARID4B* has the capability to interact with DNA sequences rich in AT base pairs, enabling it to recruit the SIN3A complex in specific regions of the genome. Once recruited, the SIN3A complex interacts with histone deacetylases (HDACs), resulting in the deacetylation of histones at the corresponding DNA locus, leading to chromatin condensation and transcriptional repression. Although we show here that *ARID4B* is essential to *KMT2A*-r ALL cells, the exact mechanistic role of *ARID4B* in this aggressive type of leukemia remains to be elucidated and further explored in the context of *KMT2A*-r ALL patient samples. From a therapeutic perspective, it would obviously be of interest to evaluate small molecule inhibitors of ARID4B in patient samples in vitro and in vivo using patient-derived xenograft (PDX) mouse models. To date, however, ARID4B inhibitors are not available.

In contrast to *ARID4B*, loss of *MBD3* was not necessarily lethal to *KMT2A*-r ALL cells, but rather inhibited leukemic cell proliferation. *MBD3* encodes a member of the methyl-CpG binding domain (MBD) protein family, which preferably binds to 5-hydroxymethylcytosine-marked genes, and has been implicated in various cellular processes, including cell differentiation, pluripotency, and cellular reprogramming [56,57,58]. Moreover, MBD3 is an essential component of the nucleosome remodeling and deacetylase (NuRD) complex, which is involved in chromatin remodeling and transcriptional regulation [57,58]. Dysregulation of *MBD3* expression or function has been observed in different human cancers, suggesting its involvement in tumorigenesis and cancer progression [59,60,61,62]. Further investigation is needed to comprehend the distinct reliance on *MBD3* in *KMT2A*-r ALL as opposed to *KMT2A*-w BCP-ALL. Unfortunately, as is the case for ARID4B, no known MBD3 inhibitors are currently available.

Finally, we found the loss of *BMPR2* to be lethal to *KMT2A*-r ALL cells. *BMPR2* encodes a member of the bone morphogenetic protein (BMP) receptor family of transmembrane serine/threonine kinases. BMP signaling is activated by binding of TGF-beta superfamily ligands and is involved in various cellular processes, including embryonic development, tissue homeostasis, cell differentiation and hematopoietic stem cell (HSC) renewal [63,64,65,66,67]. Moreover, the BMP pathway has been implicated in various cancers, including leukemia, by playing a role in tumor progression, invasion, and metastasis in various cancer types and has been recognized as a potential therapeutic strategy for cancer treatment [68]. Apart from ligand binding, activation of BMPR2 requires homodimerization or heterodimerization with BMP type 1 receptors such as BMPR1A, BMPR1B, ALK1, and ACVR1 to exert its function [69]. Subsequently, this leads to the activation of intracellular pathways, such as the SMAD signaling pathway, resulting in the regulation of target genes and cellular responses [68,70,71,72]. Interestingly, we also identified both *ACVR1* and *BMPR1A* to be potential molecular vulnerabilities in *KMT2A*-r ALL (Figure 1D,F). Hence, inhibition of BMP signaling may well induce favorable anti-leukemic effects in this aggressive type of leukemia.

In summary, the present study provides novel molecular vulnerabilities of *KMT2A*-r ALL using CRISPR-Cas9 drop-out screens targeting the human epigenome and kinome in various cell line models. While the discoveries are promising, further research and exploration is warranted, particularly in patient samples in vitro and in vivo using patient-derived xenograft (PDX) mouse models. Moreover, conducting further exploratory experiments involving knockdown (instead of knockout) techniques such as siRNA- or shRNA-mediated RNA interference, might provide valuable insights. Additionally, it would be intriguing to investigate whether the recently identified therapeutic targets are under the influence of the KMT2A fusion complex. Furthermore, exploring the potential of MENIN inhibition, which targets the interaction between MENIN and the KMT2A fusion complex [73], to effectively counteract these vulnerabilities would be of interest. Taken together, further research and exploration might lead to the development of attractive therapeutic strategies to improve the clinical outcome for patients diagnosed with *KMT2A*-r ALL.

## 4. Materials and Methods

### 4.1. Cell Culture

The pediatric *KMT2A*-rearranged B-ALL cell lines utilized in this study include SEM (*KMT2A::AFF1*^+^; ACC 546 DSMZ), KOPN-8 (*KMT2A::MLLT1*^+^; ACC 552 DSMZ) and ALL-PO [74] (*KMT2A::AFF1*^+^). ALL-PO was a gift from the lab of Prof. dr. Cazzaniga (University of Milano-Bicocca, Monza, Italy). The *KMT2A* wildtype B-cell precursor (BCP) ALL cell lines utilized include 697 (*TCF3::PBX*^+^; ACC 42 DSMZ) and NALM-6 (carrying translocation t(5;12)(q33.2;p13.2); ACC 128 DSMZ). All leukemia cell lines were cultured in RPMI-1640 medium containing GlutaMAX^TM^ supplemented with 10% fetal calf serum, 100 IU/mL Penicillin and Streptomycin, and 0.125 µg/mL Amphotericin B (Life Technologies, Carlsbad, CA, USA), at 37 °C under a 5% CO_2_-containing atmosphere. HEK293T cells (DSMZ; ACC 875) were used for virus production and maintained in Dulbecco’s modified Eagle medium (DMEM, Life Technologies) with similar supplements and cultured under similar conditions. All cell lines were routinely tested for the absence of mycoplasma and DNA fingerprinted to assure cell line authenticity.

### 4.2. Generation of Epigenome/Kinome CRISPR-sgRNA Plasmid Libraries and Lentivirus Production for CRISPR-Cas9 Knockout Screens

The epigenome and kinome sgRNA libraries were a gift from Dr. B. Evers and were previously described in Evers et al. [22]. In short, the epigenome sgRNA library consisted of 5130 sgRNAs targeting a total of 446 genes encoding epigenetic regulators, and the kinome sgRNA library consisted of 5860 sgRNAs targeting 504 genes encoding human kinases, with both libraries containing ≥10 sgRNAs per gene [22,23]. The sgRNA plasmid library was generated by cloning all sgRNAs into lentiCRISPR v2 vectors (Addgene #52961, Watertown, MA, USA) containing an U6 promoter A complete list of all gene-specific sgRNAs, as well as positive and negative (non-targeting) controls is provided in Supplementary Table S1. Virus was generated by transfection of HEK293T cells using library plasmid, MD2.G plasmid (Addgene #12259), PAX2 plasmid (Addgene #12260), and X-tremeGENE™ HP DNA Transfection Reagent (Sigma-Aldrich #XTGHP0RO, St. Louis, MO, USA). The medium was replaced by Gibco Opti-MEM (ThermoFisher #31985070, Waltham, MA, USA) the following day. Virus was harvested two days after transfection, filtered through a 0.45 µM low protein binding membrane (Millipore #HAWP04700, Burlington, MA, USA), and concentrated using vivaspin-20 columns (Sigma-Aldrich #Z614653). Concentrated virus was stored in aliquots at −80 °C for further use.

### 4.3. In Vitro CRISPR-Cas9 Knockout Screening, Sequencing, and Analysis

All cell lines were transduced with lentivirus carrying the sgRNA library via spinfection at a deliberately low Multiplicity of infection (MOI) of <0.3 to minimize the number of cells with more than one genetic editing event. We aimed to obtain at least 500 sequencing reads per sgRNA by using 1000 cells per sgRNA at the starting point of the screen. Transduction was facilitated using 4 µg/mL polybrene (Millipore #TR-1003-G). To find the optimal virus volume for achieving an MOI of <0.3, each new cell type and virus lot was tested by a titration, which was assessed by measuring cell viability (i.e., 7AAD staining) on a Cytoflex S flow cytometer (Beckman Coulter, Brea, CA, USA). 24 h after transduction, the medium was replaced to remove polybrene and puromycin selection (1 µg/mL) was initiated one day later. Then, 48 h after puromycin selection, the cells were harvested, representing baseline (Day 0) samples. The remaining surviving cells carrying the sgRNA library were maintained for another 21 days and passaged every three to four days, to harvest cells on day 21. Genomic DNA was isolated using the Trizol reagent (Life Technologies, Carlsbad, CA, USA) according to the manufacturer’s instructions. The sgRNA sequences were recovered by a first round of PCR (Biorad, Hercules, CA, USA) by using 5 µg genomic DNA to ensure the sgRNA pool complexity. The PCR primer sequences were forward 5′-ACACTCTTTCCCTACACGACGCTCTTCCGATCT NNNNNNGGCTTTATATATCTTGTGGAAAGGACG-3′ with NNNNNN as barcode and reverse 5′-GTGACTGGAGTTCAGACGTGTGCTCTTCCGATCTACTGACGGGCACCGGAGCCAATTCC-3′ as described previously [22,23,75]. The reaction mixtures were combined and purified using a QIAquick PCR Purification Kit (Qiagen, Germantown, MD, USA) according to the manufacturer’s instructions. A second PCR was performed to attach Illumina adapters and 6 bp indexing primers to the sgRNA sequences on 2ng of the purified PCR product using the forward primer 5′-AATGATACGGCGACCACCGAGATCTAC ACTCTTTCCCTACACGACGCTCTTCCGATCT-3′ and reverse primer 5′-CAAGCAGAAGACGG CATACGAGATNNNNNNGTGACTGGAGTTCAGACGTGTGCTCTTCCGATCT-3′ with NNNNNN as illumina indexing read counts as described previously [22,23,75]. Phusion Hot Start II Polymerase (ThermoFisher #F549S) was used for the PCR reactions according to the manufacturer’s manual. The amplicons were purified again and pooled equimolarly. Successful library preparation and correct amplicon length were assessed using the 2100 Bioanalyzer instrument (Agilent, Santa Clara, CA, USA). Samples were sequenced on an Illumina HiSeq2500 instrument at the Utrecht Sequencing Facility. Samples that did not pass our quality control criteria due to low read counts were excluded from the analysis. A schematic overview of the CRISPR-Cas9 knockout (KO) screen is depicted in Figure 1A.

Analysis of the data was performed on the web-based analysis platform Galaxy version 0.5.8.4 (usegalaxy.eu), using default parameters. Sequencing reads were analyzed in a model-based analysis of genome-wide CRISPR-Cas9 knockout (MAGeCK) [76], a publicly available computational tool to identify the gene’s essentiality from the CRISPR-Cas9 knock-out screening. The sgRNA read counts were normalized to the non-targeting (negative) controls using read mapping files by MAGeCK-count (Galaxy Version 0.5.8.4). We utilized the maximum-likelihood estimations (MLE) module of MAGeCK (Galaxy Version 0.5.8.1), a statistical tool that employs MLE, to determine how essential a gene is for the proliferation and/or viability of the cells, represented by a z-score which quantifies the number of standard deviations by which the normalized read counts for that gene differ from the mean. The difference in z-scores between *KMT2A*-rearranged and wild-type *KMT2A* ALL cell line models were assessed to identify genes specifically essential to either type of ALL.

### 4.4. Validation of Potential Targets by CRISPR-Cas9 Knockout Competition Assays

The candidate molecular vulnerabilities emerging from our CRISPR-Cas9 sgRNA library screens were validated by examining cell survival upon gene knockout using a competition assay [43].

The doxycyclin (DOX) inducible pCW-Cas9 vector (Addgene #50661) was utilized to express CAS9. For each target gene, one or two gRNAs were chosen from the CRISPR KO screen sgRNA libraries, complemented with one or two commercially available gRNAs against the genes of interest (Supplementary Table S2, IDT). Each gRNA was cloned into the pLKO5-sgRNA-EFS-GFP vector with the sgRNA under the U6 promoter (Addgene #57822). The lentivirus containing pCW-Cas9 or pLKO5-sgRNA-EFS-GFP expression vectors were produced, harvested, concentrated, and transduced as described above. The Cas9-expressing cell lines SEM and 697 were established through the transduction of pCW-Cas9 lentivirus, puromycin selection, and testing for Cas9 inducibility by DOX, and then transduced with pLKO5-sgRNA-EFS-GFP lentivirus containing the gRNA of interest. After four days of transduction, the GFP-positive cells were determined, mixed with non-transduced SEM or 697 cells at an equal ratio, and re-analyzed by FACS. This marked day 0 of the CRISPR KO competition assay and was set at 100% of GFP-positivity, as determined using a Beckman Coulter CytoFLEX LX Flow Cytometer with 7AAD viability dye (Biolegend, San Diego, CA, USA) to discriminate between viable and dead cells. Raw CytoFLEX data were processed using the CytExpert software version 2.3 (Beckman Coulter, Singapore). The percentage of GFP positive cells, and therefore gRNA-positive cells, were measured on days 3, 7, 14, and 21, and the percentage of GFP positive cells compared to day 0 was calculated. A schematic overview of this competition assay is illustrated in Figure 3E. On day 0 and day 21, cells were harvested, and genomic DNA was isolated using Trizol reagent (Life Technologies) according to the manufacturer’s instructions. The percentage of CRISPR KO was determined by performing PCR and Sanger sequencing of the DNA region of interest, followed by Indel quantification of the genomic locus using the Synthego ICE Analysis tool v3.0 (https://ice.synthego.com (accessed on 22 March 2022)). The primers used are listed in Supplemental Table S2.

### 4.5. Immunoblot Analysis

The levels of protein expression were determined by immunoblot analysis. For this, protein was extracted using RIPA buffer supplemented with protease inhibitors (ThermoScientific), and resolved on precast TGX™ gels and transferred to an 0.2 µm nitrocellulose membrane using a Transblot Turbo Transfer System (Bio-Rad). Blots were then probed with antibodies against MBD3 (#99169S, Cell Signaling Technology, Danvers MA, USA), ARID4B (#A302-233A-M Bethyl labs), B-actin (#ab6276 Abcam) or GAPDH (#97166S (Cell Signaling Technology). The membranes were then probed with infrared-labeled secondary antibodies IRDye 800 CW goat-anti-rabbit antibody (#926-32211, LI-COR) and IRDye 680 goat anti-mouse antibody (#926-32220, (LI-COR). Proteins were visualized using an Odyssey Infrared Imaging System (LI-COR, Lincoln, NE, USA), and protein expression was quantified using the Odyssey software Image Studio Lite version 4.0.

### 4.6. Flowcytometry Analysis

FACS analysis experiments for determination of BMPR2 expressing cells were performed on a CytoFlex Flow Cytometer (Beckman Coulter). Cells were fixed with 1% PFA before blocking with Human TruStain FcX™ (BioLegend, San Diego, CA, USA) and subsequently labeled with eBioscience™ Fixable Viability Dye eFluor™ 450 (Invitrogen, Waltham, MA, USA) to select for viable cells. Subsequently the cells were stained with BMPR2 antibody (#ab78422, Abcam, Cambridge, UK) and PE goat anti mouse IgG as secondary antibody (#405307 BioLegend) according to the manufacturer’s recommendation. Raw CytoFLEX data were processed using CytExpert version 2.3 (Beckman Coulter).

### 4.7. Statistical Analysis

Statistical significance of independent experimental replicates in the graphs were determined using two-sided Student’s *t*-tests as indicated in the figure legends. All statistical analyses were conducted using GraphPad Prism8, version 8.3.4. *p* < 0.05 was considered statistically significant.

## Data Availability

Any information required to reanalyze the data reported in this paper is available from the lead contact upon request.

## Supplementary Materials

The supporting information can be downloaded at: https://www.mdpi.com/article/10.3390/ijms241713207/s1.
