# Supplementary material for: CRISPR-Cas9 Library Screening Identifies Novel Molecular Vulnerabilities in KMT2A-Rearranged Acute Lymphoblastic Leukemia
Source: Int J Mol Sci. 2023 Aug 25;24(17):13207. doi: 10.3390/ijms241713207 (PMC10487613; doi:10.3390/ijms241713207)
Supplement: Supplementary file 1 [file ijms-24-13207-s001.zip › suppl Figure S1 controls.pdf]

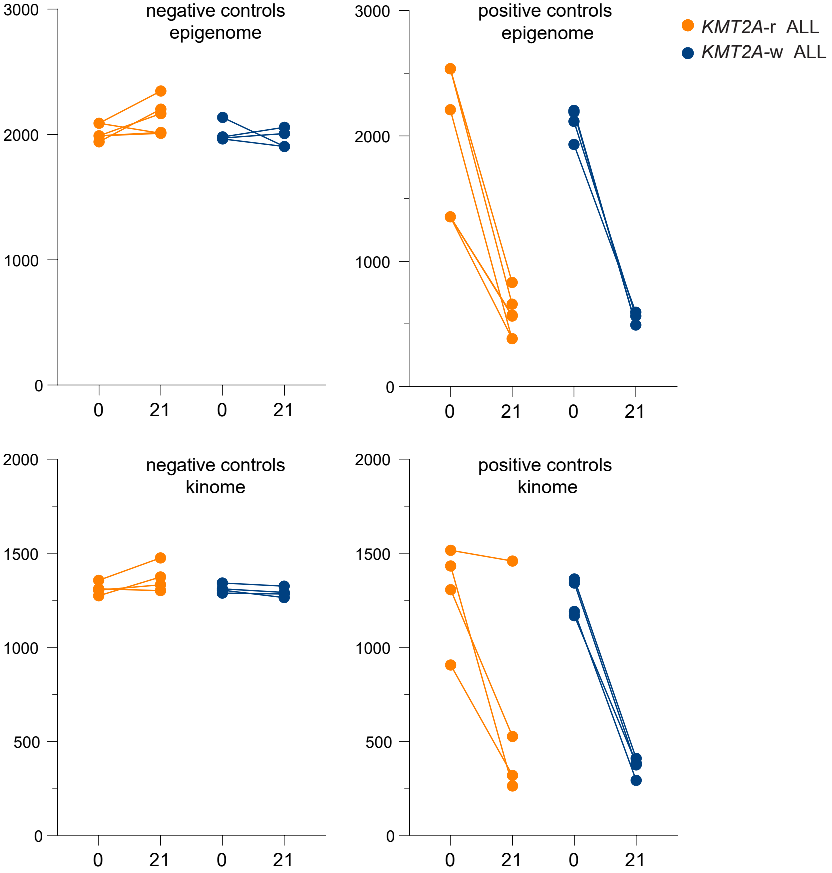

Figure S1: Evaluation of the negative and positive controls

Read counts of negative sgRNA controls, represented by 50 non-targeting control guides, and positive sgRNA controls, represented by 5 sgRNAs targeting 10 different genes esssential for human cells namely *COPB1*, *KPNB1*, *NUP98*, *PSMB2*, *PSMC4*, *PSMD6*, *PSMD11*, *RPS13*, *RPL3*, *RPL11* that were present in the CRISPR-Cas9 library screening, measured at day 0 and day 21. Each line represents a summary of the sgRNA controls of a single cell line, in orange for the *KMT2A*-r ALL cell lines and in blue for the *KMT2A-w* ALL cell lines.
