# Supplementary material for: CRISPR-Cas9 Library Screening Identifies Novel Molecular Vulnerabilities in KMT2A-Rearranged Acute Lymphoblastic Leukemia
Source: Int J Mol Sci. 2023 Aug 25;24(17):13207. doi: 10.3390/ijms241713207 (PMC10487613; doi:10.3390/ijms241713207)
Supplement: Supplementary file 1 [file ijms-24-13207-s001.zip › suppl Figure S2 CREBBP.pdf]

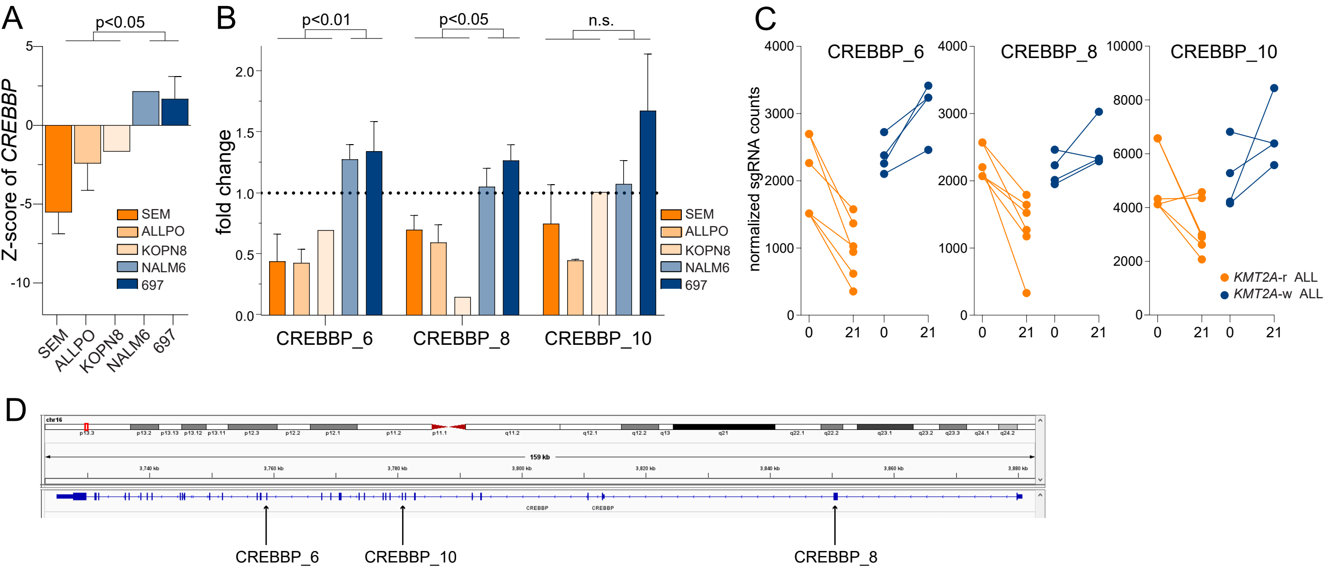


**Figure S2: Evaluation of the known *KMT2A*-r ALL vulnerability genes *CREBBP*. Related to Figure 2**

**A** Z-scores of the induvidual cell lines for CREBBP knockout. **B** Fold change at day 21 compared to day 0 of normalized read counts of sgRNAs CREBBP_6, CREBBP_8 and CREBBP_10 from the CRISPR KO screen. n=2, mean ± SEM. **C** Normalized read counts at day 0 and day 21 of sgRNAs CREBBP_6, CREBBP_8 and CREBBP_10from the CRISPR KO screen. n=2, mean ± SEM. **D** overview sgRNA locations on the CREBBP gene
